# Spatial analysis of placentae during congenital cytomegalovirus infection suggests distinct cellular profiles in immune and trophoblast cells: an exploratory study

**DOI:** 10.3389/fimmu.2026.1885923

**Published:** 2026-10-12

**Authors:** Elise Sintim-Aboagye, Huy Quang Quach, Indrani Mukherjee, Will Sherman, Katelyn T. Nguyen, Neil Ferland, Sheila Farnan, Kamila Otrubova, Namisha Verma, Dawn Littlefield, Sohan Punia, Erica Johnson, Mark Blackstad, Mark R. Schleiss, Andrew P. Norgan, Clive M. Gray, Elizabeth Ann L. Enninga, Rana Chakraborty

**Affiliations:** 1 Department of Obstetrics and Gynecology, Mayo Clinic, Rochester, MN, United States; 2 Mayo Clinic Vaccine Research Group, Department of Internal Medicine, Mayo Clinic, Rochester, MN, United States; 3 Department of Pediatrics, Division of Infectious Diseases, University of Miami, Miami, FL, United States; 4 Department of Quantitative Health Sciences, Mayo Clinic, Rochester, MN, United States; 5 Department of Pediatric and Adolescent Medicine, Mayo Clinic, Rochester, MN, United States; 6 Department of Microbiology, Biochemistry, and Immunology, Morehouse School of Medicine, Atlanta, GA, United States; 7 Department of Pediatrics, Division of Pediatric Infectious Diseases, University of Minnesota Medical School, Minneapolis, MN, United States; 8 Department of Laboratory Medicine and Pathology, Mayo Clinic, Rochester, MN, United States; 9 Division of Immunology, Department of Biomedical Sciences, Biomedical Research Institute, Stellenbosch University, Cape Town, South Africa

**Keywords:** congenital cytomegalovirus, immune cells, omics, placenta, trophoblast

## Abstract

**Introduction:**

Human cytomegalovirus (CMV) is the leading infectious cause of congenital birth defects worldwide. The placenta is a major route of vertical transmission, and both immune and trophoblast cells are susceptible to CMV infection. This study aimed to spatially characterize gene and protein expression changes associated with CMV infection in the human placenta.

**Methods:**

In this exploratory study, CMV+ and control placentae were analyzed using the NanoString GeoMx Digital Spatial Profiler to assess compartment-specific transcriptomic and proteomic profiles in trophoblast and immune cell populations.

**Results:**

CMV+ placentae showed increased immune activation in both trophoblast and immune cells, including pathways related to immune-cell proliferation, differentiation, lymphopoiesis, and T-cell development. Infected placentae also showed altered tissue repair and remodeling pathways, along with changes in proapoptotic proteins and cell death-associated transcripts.

**Discussion:**

Given the limited sample size, these findings are descriptive and hypothesis-generating. They provide preliminary insight into CMV-associated placental immune dysregulation and identify candidate pathways for investigation in larger, prospective studies.

## Graphical Abstract

**Figure:**
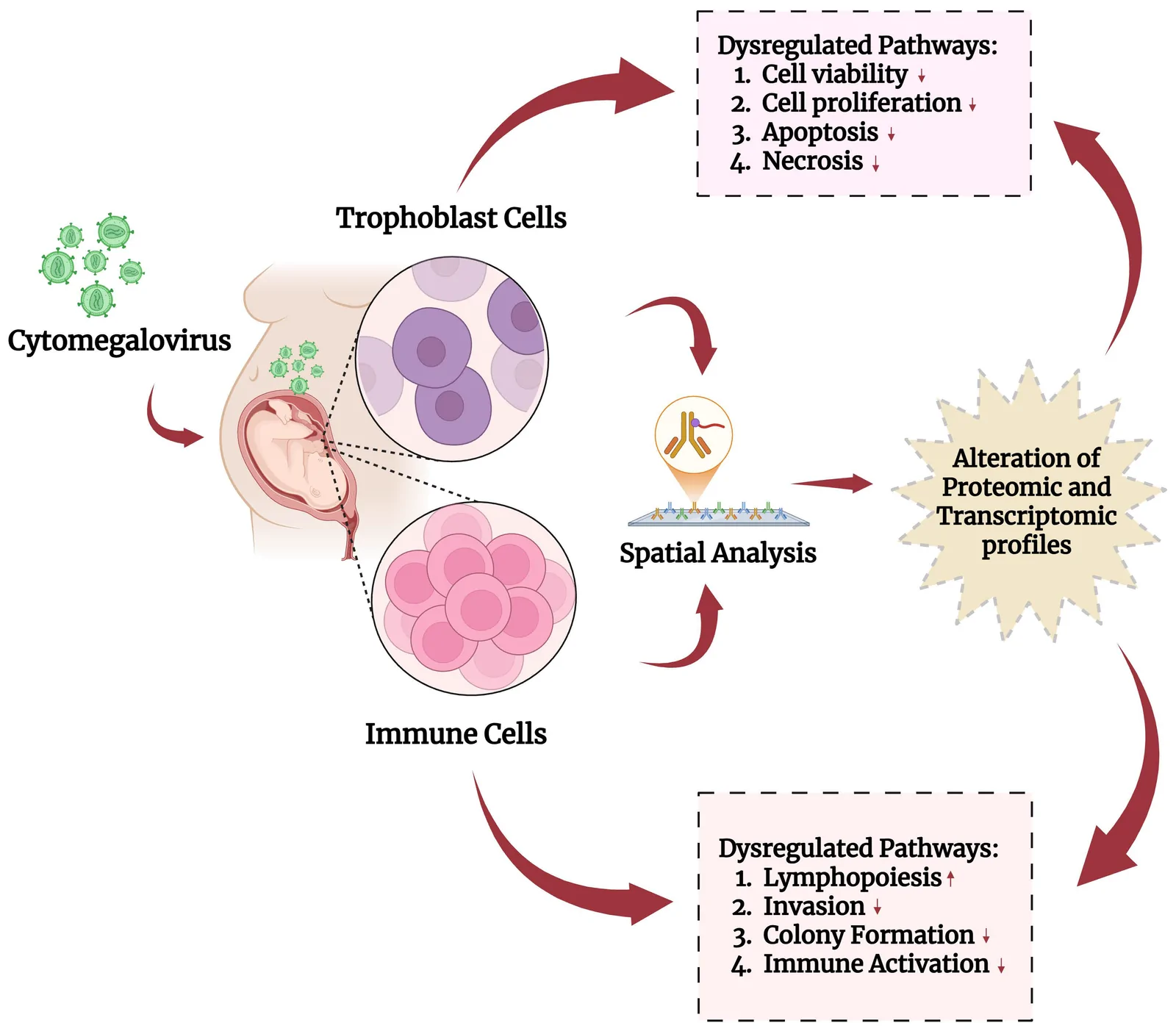
Spatial-omic profiling reveals compartmentalized immune responses in CMV+ placentae, shedding light on mechanisms of vertical transmission and immune evasion.

## 1 Introduction

Congenital CMV (cCMV) infection is the most common infectious cause of birth defects globally, affecting approximately one million infants annually (1, 2). In the United States, the Centers for Disease Control and Prevention estimates that cCMV infection occurs in approximately 1 in 200 live births and nearly one in three children are infected with CMV by age five (3). Roughly 10% of neonates with cCMV are symptomatic at birth, often presenting with multi-system involvement that contributes to substantial morbidity and mortality (4). Furthermore, even asymptomatic infants can develop long term complications, including sensorineural hearing loss, impaired psychomotor development, and impaired growth (5–8).

Preventive strategies, particularly those based on vaccination, may substantially reduce disease burden (9). However, progress has been hindered by challenges in vaccine development; largely due to a poor understanding both of CMV pathogenesis (10) and the immunological dynamics of the maternal-placental-fetal interface (11). Transmission of CMV can occur at various stages: vertically during intrauterine fetal development, perinatally via exposure to blood and vaginal secretions, and postnatally through breastfeeding (12, 13). Among these, vertical transmission via the maternal blood and placenta is the primary and most clinically relevant route for cCMV infection (14).

Previous studies have shown that CMV can infect multiple placental cell types including cytotrophoblasts (CTB), syncytiotrophoblasts (STB), and decidual immune cells leading to inflammation, barrier dysfunction, and altered cytokine signaling (15). However, prior work has largely relied on *in vitro* models and have not applied spatial resolution and cell-type specificity. In this study, we investigated the effects of CMV infection on placental tissue using spatial multi-omics approaches. By leveraging the NanoString Digital Spatial Profiler (DSP), we aimed to delineate gene and protein expression profiles of trophoblast and immune cells within distinct placental compartments. Our key research question was: How do gene and protein expression differ by cell type in CMV-infected placentae?

## 2 Methods

### 2.1 Ethics statement

The Institutional Review Board (IRB) of Mayo Clinic approved this study (IRB #20-012379).This retrospective study utilized clinical residual tissues from patients who provided Minnesota Research Authorization. Placentae for each group were selected by searching the pathology database. CMV case inclusion required confirmation of a congenital CMV diagnoses based on antenatal ultrasound, serology, PCR, and by clinical signs and symptoms in postnatal life. Cases of fetal demise or those where vertical transmission of CMV to the infant was not definitive were excluded from selection. Only participants who underwent Cesarean delivery were selected to minimize the possible effects from inflammation during a vaginal delivery.

### 2.2 Placental tissue samples

Formalin fixed paraffin embedded (FFPE) placental tissue from 7 participants who underwent Cesarean section were obtained from the Mayo Clinic Tissue Repository and sectioned by the Mayo Clinic Pathology Core. Clinical confirmation of CMV in the placenta was achieved using immunohistochemistry (IHC; clones CCH2 + DDG9, Dako) which targets the early immediate early antigen (IEA) and early antigen (EA). Three samples were from pregnancies with a confirmed diagnosis of primary CMV infection during pregnancy, and four were obtained from uncomplicated pregnancies undergoing elective Cesarean section with normal placental histology and no documented underlying infection.

### 2.3 Detection of CMV viral DNA in affected placental samples

Polymerase Chain Reaction (PCR) was performed to detect the presence of CMV DNA in placental tissue from infants diagnosed with congenital infection as it has been reported to detect cCMV cases with more precision than IHC staining (16). Real-time PCR was performed in triplicate using a laboratory SOP as previously described (17, 18) with modifications. The primers used for UL83 detection were: forward UL83_TM857R (GGACACAACACCGTAAAGC); reverse UL83_TM1138R (GTCAGCGTTCGTGTTTCCCA); and probe CFR610-CCCGCAACCCGCAACCCTTCAT-BHQ2. The primers used as a control for human DNA recovery (NRAS detection) were: forward (GCCAACAAGGACAGTTGATACAAA); reverse (GGCTGAGGTTTCAATGAATGGAA); and probe, FAM-ACAAGCCCACGAACTGGCCAAGA-BHQ1.

A standard curve for the UL83 control was generated using 10-fold dilutions from 10^6^ to 10 copies/µL of a plasmid (UL83 fragment cloned in pCR2.1, using primers UL83_TM857F and UL83_TM1138R). The standard curve for NRAS was generated using five 10-fold dilutions starting with 200,000 to 20 pg/µL of human genomic DNA (Roche).

### 2.4 Transcriptomic analysis by GeoMx digital spatial profiling

FFPE tissues were baked at 60 °C for 3 h, then deparaffinized and rehydrated prior to antigen retrieval with Tris EDTA. RNA targets were exposed with 1 μg/mL Proteinase K followed by fixation with 10% formalin. Slides were then incubated overnight with an RNA probe mix (GeoMx Human Whole Transcriptome Atlas; Brucker Spatial Biology, Seattle, WA) The next day, post-fixing steps were performed before staining with the following morphology markers: anti-CD45-AF594 (2B11+PD7/26, 5 µg/mL), anti-PanCK-AF532 (AE1+AE3, 2 µg/mL) and SYTO13-FITC (Brucker, 1 µg/mL). Antibodies were purchased from Novus (Centennial, CO). Slides were loaded onto the GeoMx DSP instrument, and for each tissue sample, a total of four regions of interest (ROIs) were collected from the villi. Each ROI was subdivided into areas of illumination (AOIs) based on fluorescent cell-specific markers CD45 and PanCK. Serial UV illumination of each compartment was used to sequentially collect the mRNA probe barcodes from each cell type. Each sample plus non-template controls (NTCs) were uniquely indexed using the i5 × i7 dual-indexing system (Illumina, San Diego, CA). The libraries were then sequenced using the Illumina NextSeq2000 P2 (2x50) for a maximum of 800M aligned reads.

### 2.5 Proteomic profiling by NanoString GeoMx

The method recommended for GeoMx DSP (Brucker) was used in this study as per the manufacturer’s protocol. Briefly, 5 µm sections of FFPE placental tissue were baked for 3 h at 60°C before tissue deparaffination and rehydration. Antigen retrieval was completed using citrate buffer. Slides underwent a blocking step with Buffer W (Brucker) for 1 h at room temperature and were then incubated overnight with the three fluorescent antibodies listed above and a panel of 49 oligonucleotide (oligo)-conjugated antibodies at 4°C. The slides were scanned on the GeoMx DSP, and twelve ROIs (corresponding to 144 AOIs) were selected per slide. We selected eight ROIs from the villi and four ROIs from the decidua. After ROI selection, UV light cleaved the photocleavable oligos off the antibodies and were transferred onto a 96-well plate for hybridization and quantification on the nCounter (Brucker).

### 2.6 Immunohistochemistry

Placental tissue samples were washed with PBS and fixed in 4% buffered formalin at room temperature. Paraffin embedment was done, and 5 µm thick sections were cut. The sections were deparaffinized, rehydrated, and microwaved for 20 min in Tris–EDTA buffer (pH 9.0) to retrieve antigens and blocked with 3% H_2_O_2_ for 15 min. Non-specific reactivity was blocked using BlockAid™ Blocking Solution (Invitrogen). Sections were incubated with rabbit anti-Bcl-2 antibody (dilution: 1:1000; ab182858), or rabbit anti-cleaved caspase 3 (CC3) antibody (dilution: 1:1000; ab32351), at 4 °C in a humid chamber for 12–14 h. Sections were then incubated with fluorophore-conjugated anti-rabbit secondary antibody (dilution: 1:1000; ab6721) at room temperature for one hour, washed thrice with 0.1 M TBS, and mounted with DPX mounting medium. The sections were observed under a fluorescence microscope, using appropriate filter sets. Staining was quantified using QuPath. Five equal sized fields of view (FOVs) were selected from each slide. Cell detection and positive cell detection were used to determine positive cells (DAB positive) within each FOV and averaged for each tissue.

### 2.7 Statistical analysis

Metadata merging was conducted using the DSP Analysis Suite. Subsequent statistical analysis was performed using R (v4.2.2). Raw spatial proteomics count data was processed through quality control and normalization strategies that were recommended by the manufacturer. Quality control was based on Field of View Registration, Minimum Surface Area of ROIs, Minimum Nuclei Count within ROIs, and Binding Density. Proteomic data was normalized using housekeeping proteins S6, Histone H3, and spike-in negative nCounter hybrid control. GeoMx spatial transcriptomics sequencing raw Illumina counts were filtered using standard QC threshold settings as recommended by the manufacturer; batch effects were investigated using limma (v3.60). Counts were normalized to the upper quartile. Processing count data was done using GeomxTools (v3.2.0) and NanoStringNCTools (v1.6.1). Comparisons for individual gene expression were measured as a log2 fold-change (FC). A linear mixed model was used to fit the normalized RNA count data to identify differentially expressed genes (DEGs); a similar construction was used for normalized Protein count data. The mixed model accounts for intrasample variability via the random effect term. The adjustment method to control false discovery rate (FDR) for multiple testing used in this study was the Benjamini and Hochberg method (19). The analysis was carried out using the lme4 (v1.1.31) and stats packages (v4.1.2) with emmeans (v1.8.4.1).

## 3 Results

### 3.1 Confirmation of CMV-infection and subject demographics

FFPE placental tissue was obtained from the Mayo Clinic Tissue Registry. For transcriptomic and proteomic analyses, tissue samples from women documented to have primary CMV infection (CMV^+^) during pregnancy, confirmed by clinical IHC staining.

Placental CMV infection was further established by RT-PCR for UL83 as a measure of viral load (**Supplementary Table 1**). Six samples (1–6) were found to be negative while the next six samples (7–12) were all found to be positive. Samples 7–11 were of low average viral load (1.53 to 5.20 E + 01 copies/µg). Samples 7–10 all had (3 of 3) positive replicates. Sample 11 had (2 of 3) positive replicates so the average viral load was calculated using the average of the positive replicates. Sample 12 had a much higher average viral load (1.25E + 04 copies/µg) than the other positives. All the test samples (1–12, 69,84) were also found to contain sufficient amplifiable human genomic (hg) DNA as indicated by the NRAS qPCR assay. The CMV standard and positive control amplified and quantified as expected for the CMV (UL83) assay (20). The human genomic DNA standard also amplified as expected for the NRAS assay.

Overall, we included 3 CMV^+^ tissues and 4 CMV^-^ samples. The selected samples had comparable maternal ages, Body Mass Index (BMI), and neonatal APGAR scores between groups (**Table 1**). Neonates born to patients with CMV infection had a lower median birth weight than those from control subjects (*p* = 0.05). Hematoxylin and eosin images, and CMV^+^ IHC staining, are shown in **Supplementary Figure 1**. Representative immunofluorescent images illustrate the villous (**Figure 1A**) and decidual (**Figure 1B**) ROIs collected from a CMV^+^ sample.

**Table 1:** Patient characteristics.

Criteria	CMV+ (n=3)	Control (n=4)	Total (n=7)	P-value
Maternal Age (years)	28 (24-32)	28.8 (23-38)	28.4 (23-38)	0.7714
Pre-gravid BMI (kg/m^2^)	34.3 (28.7-41.0)	27.4 (19.0-20.4)	30.3 (19.0-41.0)	0.400
Gestational Age (weeks + days)	32+2 (31+1 – 34+6)	37+6 (33+5 – 39+2)	35+6 (31+1 – 39+6)	0.1714
Birthweight (kg)	1.64 (1.04-2.05)	2.99 (2.4-3.30)	2.41 (1.04-3.30)	0.0571
APGAR Score at 5 mins	8 (6-9)	8 (6-9)	8 (6-9)	>0.9999

**Figure 1:**
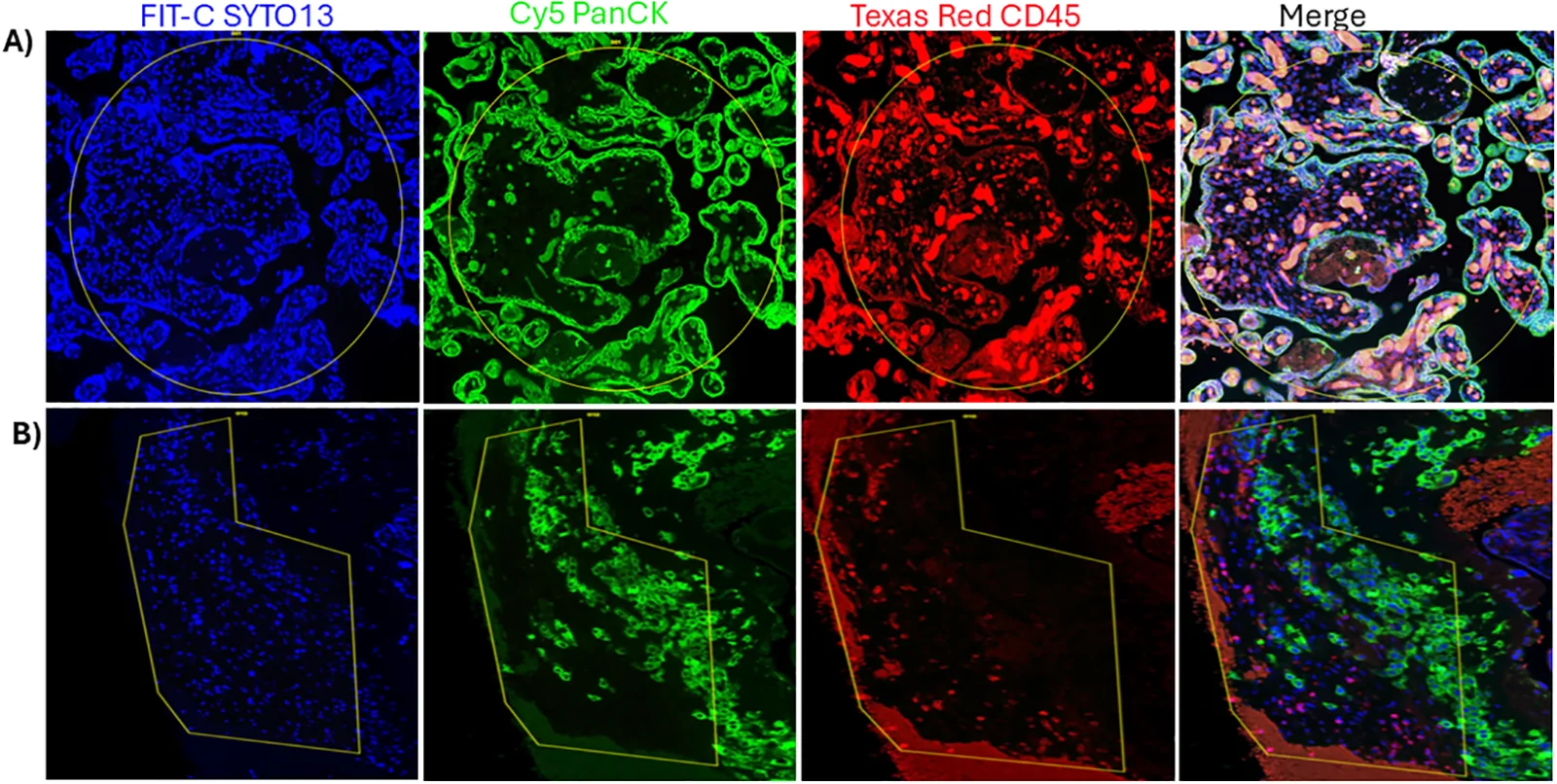
Representative images of a CMV^+^ placental ROI from the GeoMx digital spatial profiler. CD45^+^ cells are shown in red, PanCK^+^ cells are shown in green, and nuclei (SYTO13) shown in blue. **(A)** Villous tissue. **(B)** Decidual tissue.

### 3.2 Altered gene expression in CD45^+^ immune cells infected with CMV

Following successful normalization (**Supplementary Figure 2**), we evaluated the transcriptomic profiles of immune cells within CMV^+^ versus CMV^-^ placental tissue to identify which genes were differentially expressed. In CD45^+^ immune cells, we observed 60 DEGs, of which 58 genes were significantly upregulated, and 2 genes significantly downregulated in CMV-infected placental tissue compared to no CMV infection (**Figure 2A**; **Supplementary Figure 3**). In contrast to CMV^-^ tissue, DEGs in CMV-infected CD45^+^ immune cells were enriched in numerous immune system activation pathways using Ingenuity Pathway Analysis (IPA; Qiagen), including immune cell proliferation and differentiation, lymphopoiesis, and T cell development (**Figure 2C**). In addition, pathways implicated in tissue repair and remodeling, such as apoptosis of fibroblasts, failed to be upregulated in CMV^+^ placental tissue when compared to CMV^-^ tissue. Together, these data suggest that processes critical for healthy pregnancy outcomes are altered at the transcriptional level during CMV infection.

**Figure 2:**
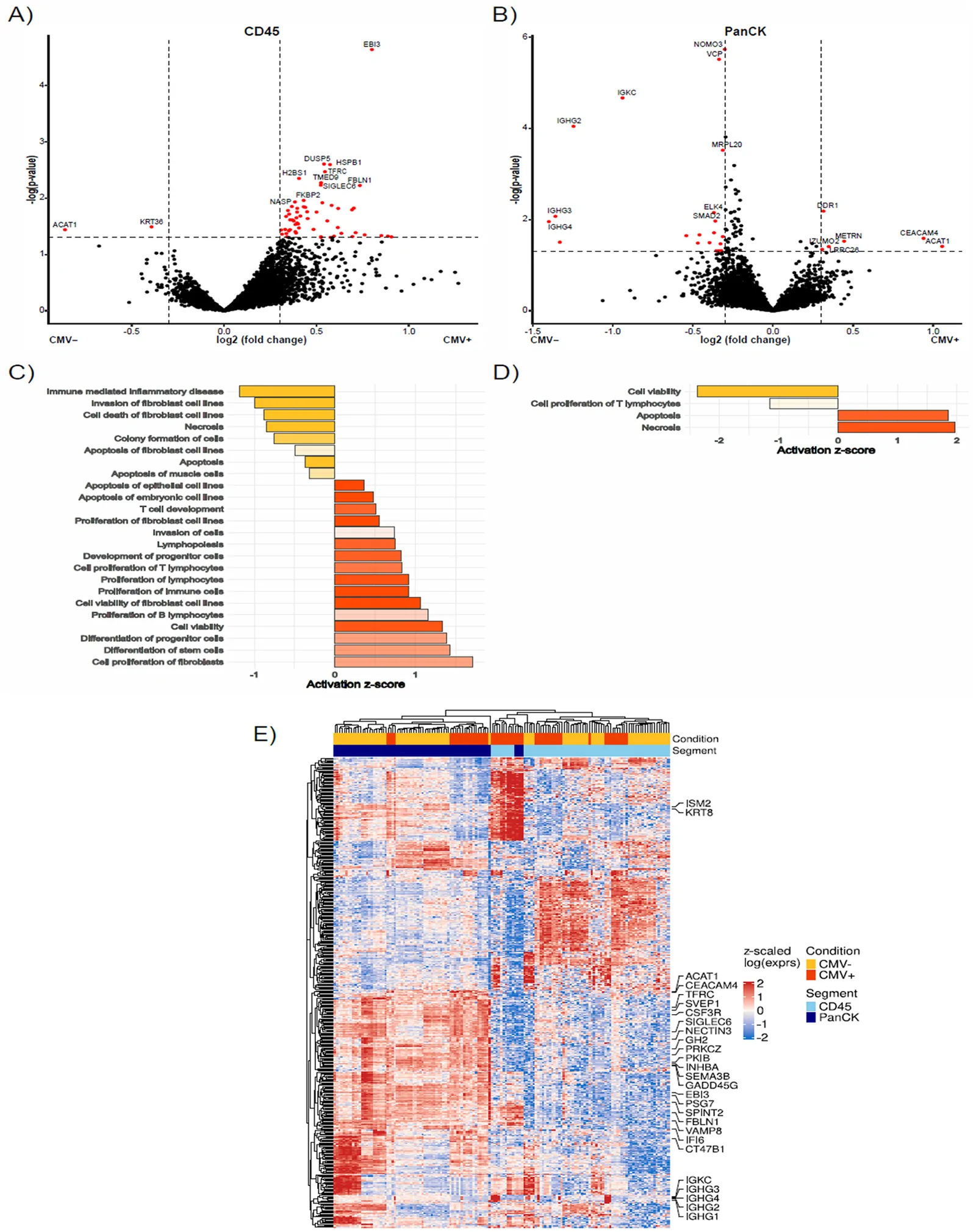
Top transcriptional pathways in CMV infected placentae. **(A)** The differential expression results between CMV+ and CMV- conditions for all CD45 AOIs. A maximum of 10 top DE genes per condition are depicted per direction. **(B)** The differential expression results between CMV+ and CMV- conditions for all PanCK AOIs. A maximum of 10 top DE genes per condition are depicted per direction. **(C)** Top pathways for CD45+ AOIs in CMV− (yellow) or in CMV+ (orange) samples. **(D)** Top pathways for PanCK+ AOIs showing increased activation in CMV− (yellow) or in CMV+ (orange) samples. Non-significant pathways and those with no Activation z-score defined by Qiagen IPA were excluded. **(E)** Heatmap of top 500 highly variable genes (HVGs) of log2 normalized counts showing distinct hierarchical clustering, grouping trophoblast and immune regions but also showing distinct clustering by CMV-status within cell type. Several HVGs which were also differentially expressed with absolute-log fold change > 0.5 and p-value < 0.05 included.

### 3.3 Altered gene expression in PanCK^+^ trophoblast infected with CMV

Changes in trophoblast gene expression across all subtypes were analyzed between CMV^+^ and CMV^-^ placentae using PanCK. We identified 47 DEGs in trophoblast from CMV-infected placentae compared to non-infected controls, with 40 genes downregulated, and 7 genes upregulated (**Figures 2B, D**). The corresponding IPA of these trophoblast cells revealed that when compared to CMV^-^ samples, trophoblast exposed to CMV demonstrated increased expression of pathways related to apoptosis and necrosis. Furthermore, pathways implicated promoting cell viability failed to be transcriptionally upregulated in CMV^+^ when compared to CMV^-^ trophoblast populations. Lastly, hierarchical clustering of the top 500 highly variable genes (HVGs) based on log2 normalized counts revealed distinct grouping by trophoblast and immune regions, as well as separate clustering by CMV status within each cell type, with several of these HVGs also differentially expressed (absolute log fold change > 0.5, p < 0.05) (**Figure 2E**).

### 3.4 Upregulation of immune activation proteins and downregulation of cell death proteins in CD45^+^ immune cells during CMV infection

Phenotypes of CD45^+^ cells in the placental villi and decidua were analyzed to determine if there are spatial differences in these compartments. There was a significant increase in immune cells within the villous tissue having an activated phenotype during CMV infection compared to CMV^-^ tissue (**Figure 3A**; **Supplementary Figure 4**). These activation markers were CD27 and CD44. Conversely, BAD, BCLXL, BIM, p53, GZMA, and BCL6, were downregulated in immune cells in the villi during CMV infection compared to non-CMV-exposed tissue. Within CMV^+^ decidual tissue, we observed an increase in CD27 and downregulated expression of proteins involved in apoptosis BCLXL, BIM, p53, PARP, GZMA, and BCL6 compared to CMV^-^ decidual tissue (**Figure 3B**). Although these dysregulated protein expression trends are similar between the CMV^+^ villi and deciduae, we sought to identify differences in individual protein expression between the two tissues. We determined that overall, villous tissue exhibited a significantly higher abundance of BAD, BCLXL, and p53, but a significantly lower abundance of CD44 when compared to decidual tissue (**Figure 3C**). While apoptotic marker expression was decreased overall in the decidua, cell death protein expression was still significantly downregulated in this compartment in CMV^+^ compared to CMV^-^ tissue (**Figure 3D**). Overall, these data indicate that CMV exposure may cause dysregulated expression of apoptotic proteins, and that the immune cells in the villi spatially expressed cell death markers at higher levels than in the decidua.

**Figure 3:**
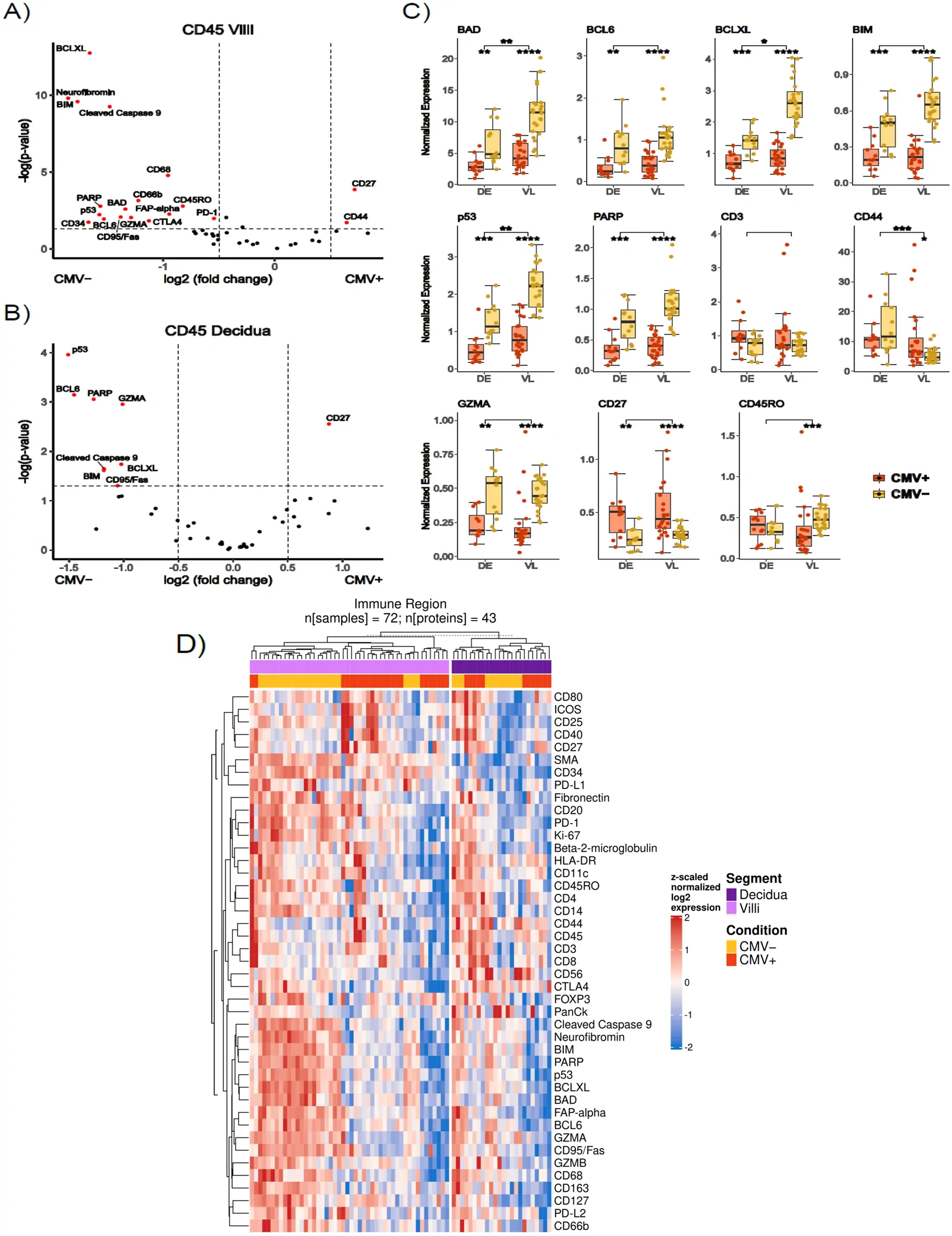
Differences in proteins altered on CD45+ cells of placentae. Volcano plots for differential spatial proteomic analysis of normalized GeoMx DSP nCounter for protein fluorescence intensities. Positive fold changes represent upregulation in CMV +. **(A)** Differential expression for AOIs annotated as being CD45+ from fluorescence masking in the villi. **(B)** Differential expression for AOIs annotated as being CD45+ from fluorescence masking in the decidua. **(C)** Normalized expression box plots for several top markers, subset by ROI location to decidua (DE; n=24 ROIs) and villi (VL; n=48 ROIs) or CMV+ (n=36 ROIs) and CMV- (n=36 ROIs). Wilcoxon Rank Sum test with the top bar comparing differences between tissue location and the below bar compares CMV+ and CMV− results. *p ≤ 0.05; **p ≤ 0.01; ***p ≤ 0.001; ****p ≤ 0.0001. **(D)** Heatmap of protein expression profile within the Immune ROIs; complete-linkage hierarchical agglomerative clustering of log2 normalized counts shows distinctive grouping by condition and partial association by tissue region.

### 3.5 Protein expression changes in trophoblast infected with CMV

Protein expression in PanCK^+^ trophoblast cells, which would include all 3 major subtypes (CTBs, STBs and extravillous trophoblasts) from CMV^+^ placental tissue was analyzed. The trophoblast populations from CMV^+^ villous tissue displayed significant downregulation of BAD, BCLXL, BIM, p53, PARP, GZMA, and BCL6 compared to uninfected villi (**Figures 4A, B**). We also observed a discordance in the expression of immune activation markers when compared to CMV^-^ villi, the CMV^+^ villi expressed higher levels of CD44 and CD27, but lower levels of CD3 and CD45RO. However, overall protein expression in the villi, with or without CMV exposure, was higher than in the decidua (**Figure 4C**). Overall, trophoblast phenotypes during CMV infection exhibited downregulated apoptotic and differentiation markers.

**Figure 4:**
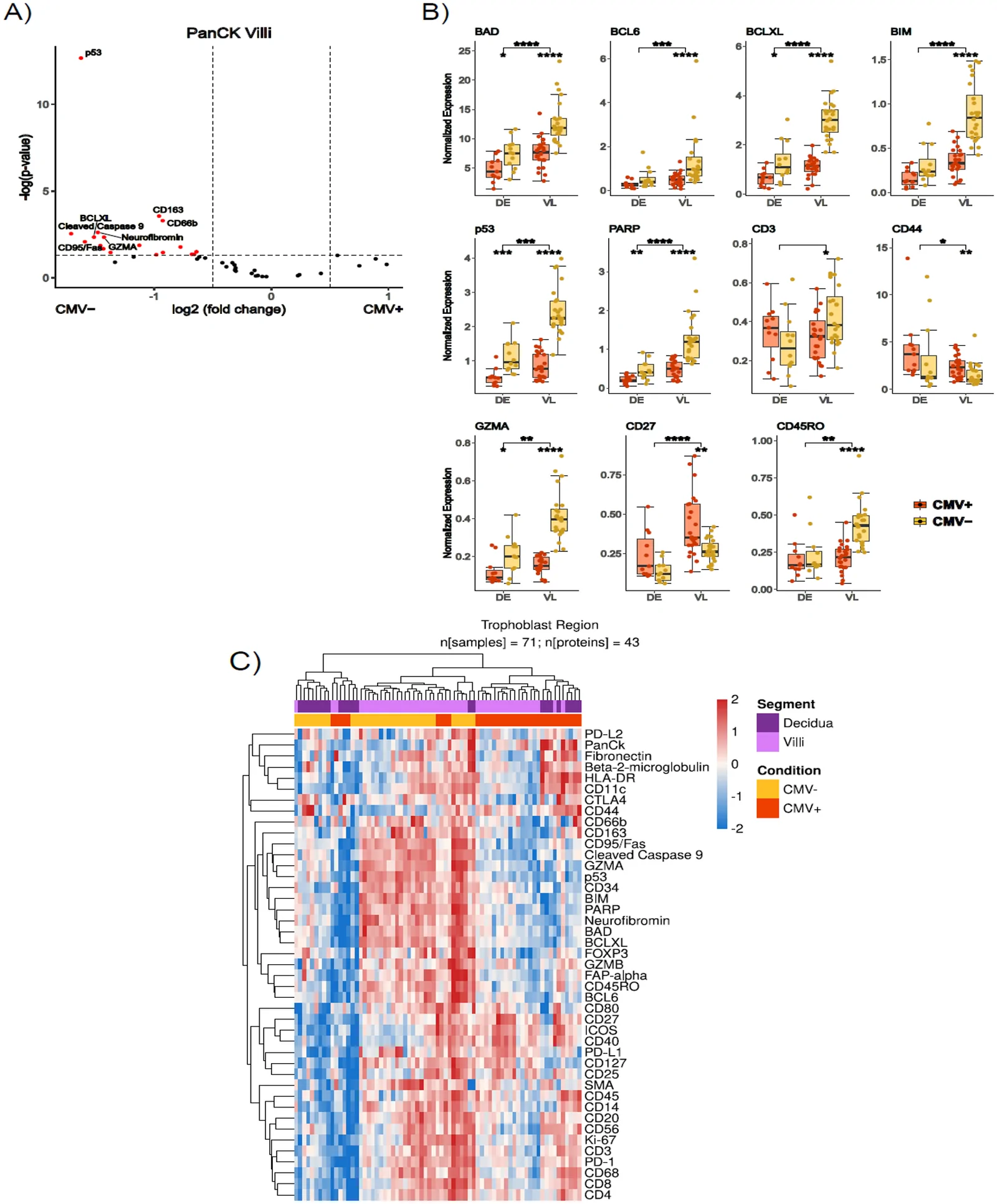
Altered protein expression in PanCK^+^ cells of placentae. Volcano plots for differential spatial proteomic analysis of normalized GeoMx DSP nCounter for protein fluorescence intensities. Positive fold change represents upregulation in CMV +. **(A)** Differential expression for AOIs annotated as being PanCK+ from fluorescence masking in the villi. **(B)** Normalized expression box plots for several top markers, subset by ROI location to decidua (DE; n=24 ROIs) and villi (VL; n=48 ROIs) or CMV+ (n=36 ROIs) and CMV- (n=36 ROIs). Wilcoxon Rank Sum test with the top bar comparing differences between tissue location and the below bar compares CMV+ and CMV− results. *p ≤ 0.05; **p ≤ 0.01; ***p ≤ 0.001; ****p ≤ 0.0001. **(C)** Heatmap of protein expression profile within the Trophoblast ROIs; complete-linkage hierarchical agglomerative clustering of log2 normalized counts shows tight groupings by both condition and tissue region.

### 3.6 Detection of BCL-2 and CC3 in CMV^+^ and control tissue

Immunohistochemistry was performed to determine the presence of BCL-2 and Cleaved caspase 3 (CC3) in control and CMV^+^ tissues (**Figure 5**). Results indicate that CMV+ tissues contained more positive cells per field of view (FOV) of CC3 than control tissues (**Figure 5A**; p=0.0267). DAB staining was specific to lymphocyte cells within the villi, and not to the syncytiotrophoblast population. Conversely, CMV+ tissues had significantly less BCL2 cell positivity than control per FOV. (**Figure 5B**; p=0.0235). DAB staining was much more pronounced on the syncytiotrophoblast layer than those cells within the villi.

**Figure 5:**
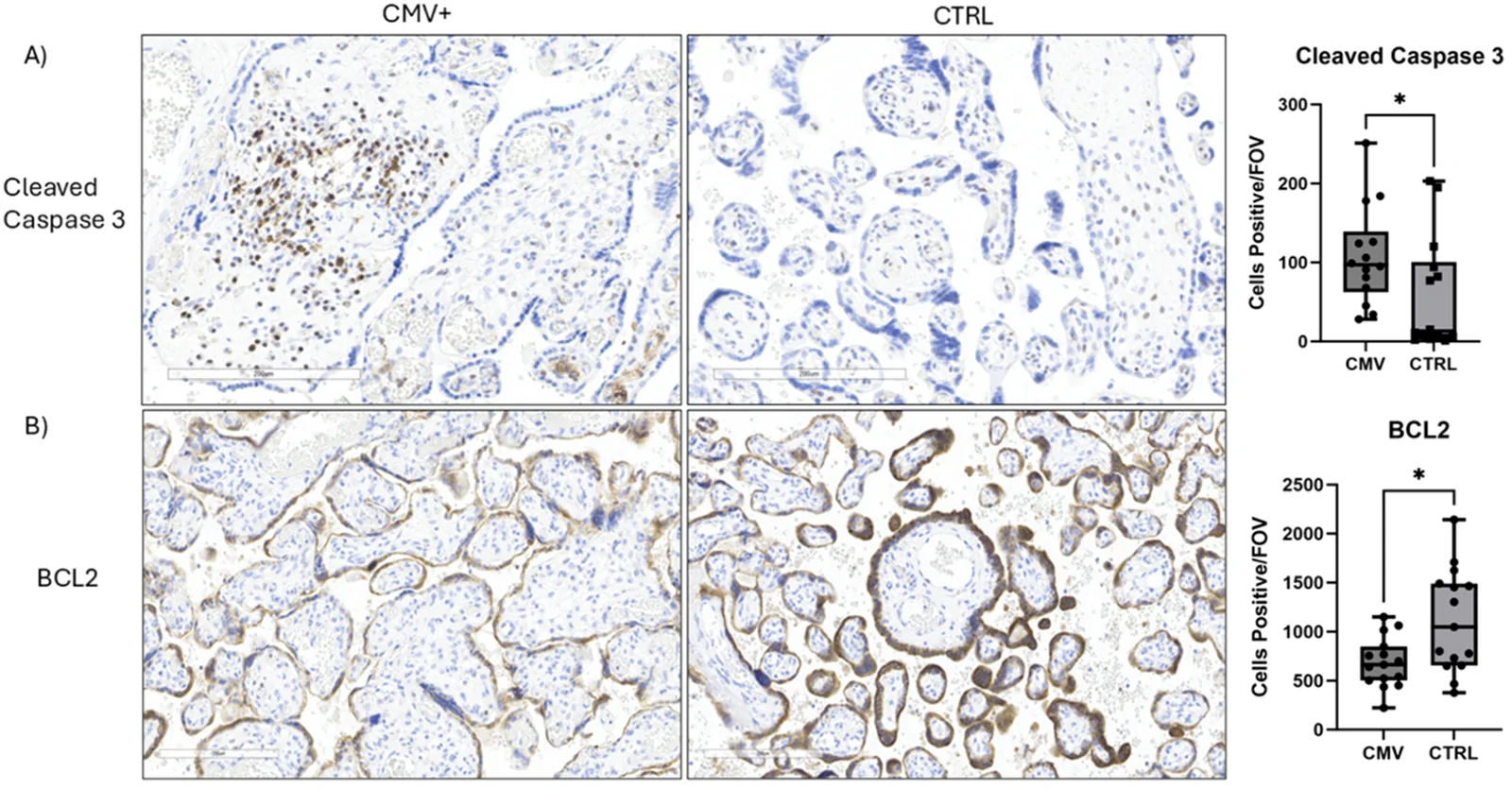
CMV induced changes in apoptotic protein expression on cells of the placenta. Representative immunohistochemistry images from control and CMV^+^ tissues stained for **(A)** cleaved caspase 3 (CC3) or **(B)** BCL2 with DAB+ cell quantification by QuPath (n=15 total FOV). Scale bars at 200 microns. Mann-Whitney Test with a *p-value ≤0.05.

### 3.7 Concordance between transcriptomic and proteomic analyses of Both CD45^+^ immune cells and PanCK^+^ trophoblast in CMV^+^ vs. CMV^-^ tissue

To validate whether there were correlations between transcriptomic and proteomic profiling across CMV^+^ and CMV^-^ tissue, we compared the gene probe quantifications (GeoMx Whole Transcriptome Atlas [WTA]) against the corresponding normalized protein expression intensities (GeoMx Human Protein Assay for nCounter [Cell Death, Immune Activation Status, or Immune Cell Profiling panels]). In CD45^+^ immune cell AOIs (**Figure 6A**) and PanCK^+^ trophoblast AOIs (**Figure 6B**), Spearman correlation analyses revealed positive associations between transcriptional and protein expression across both CMV^-^ (r = 0.51 and *p* = 0.013 in CD45^+^ cells; r = 0.51 and *p* = 0.013 in PanCK^+^ cells) and CMV^+^ (r = 0.62 and *p* = 0.015 in CD45^+^ cells; r = 0.45 and *p* = 0.029 in PanCK^+^ cells) placental tissue, indicating an overall agreement across the two modalities. Next, we evaluated the directionality and magnitude of differential expression for the proteomic and transcriptomic analyses. Within CMV^+^ tissue, most significantly altered pathways in CD45^+^ (**Figure 6C**) or PanCK^+^ (**Figure 6D**) cells showed up- or down-regulation consistently across both modalities. This demonstrates the downregulation of GZMB, CD68, BCL2L1, and PTPRC in CD45^+^ cells, and PTPRC, TP53, KRT8 in PanCK^+^ cells at both the RNA and protein expression levels. However, a subset of pathways showed discordant transcriptional and protein expression in CD45+ cells (CD27, PARP1, KRT8) and in PanCK^+^ cells (CD163, NF1, BCL2L1), highlighting potential post-transcriptional regulation or reduced protein stability during CMV infection.

**Figure 6:**
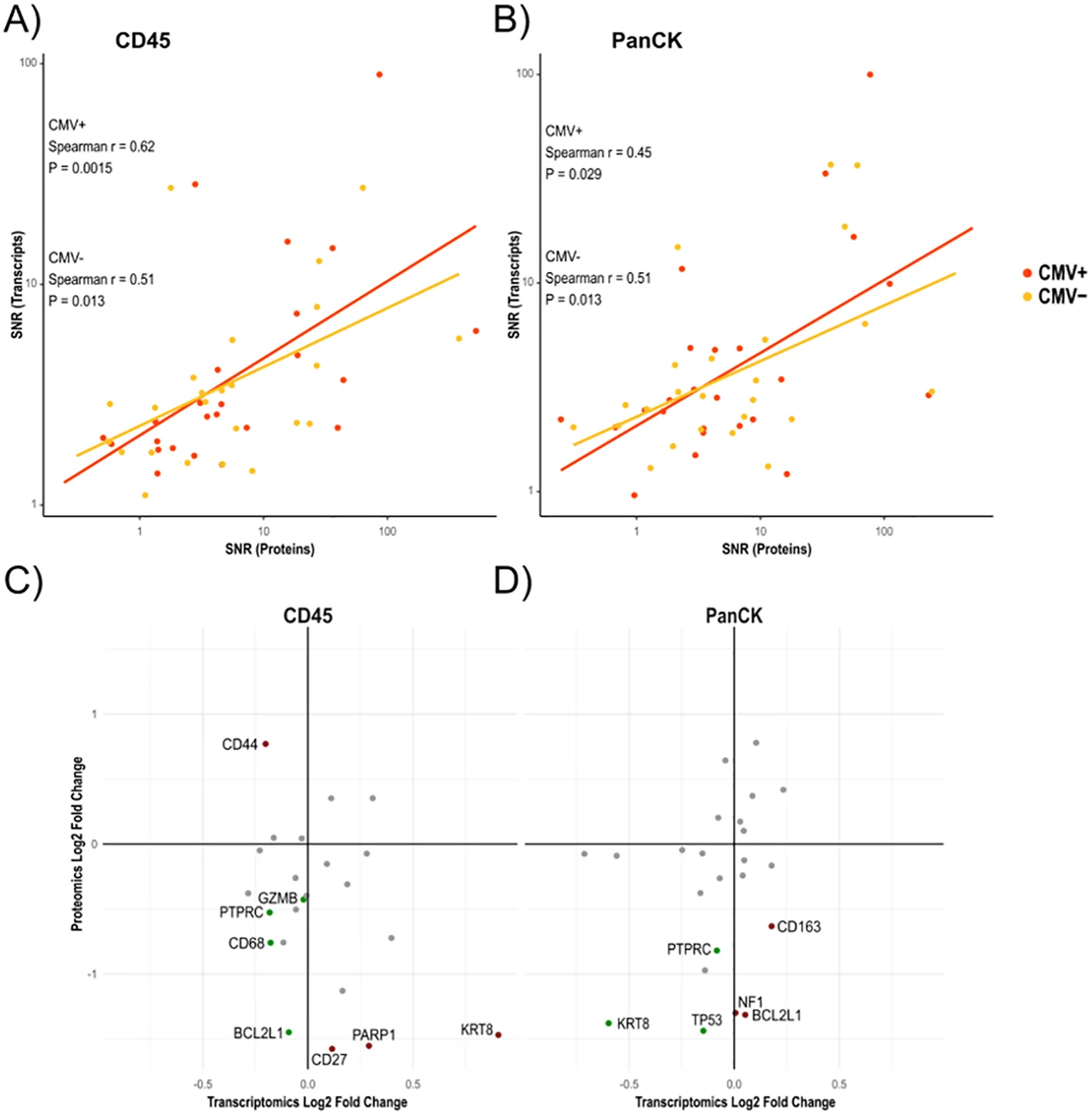
Correlation between transcriptomic and proteomic data in CMV^-^ and CMV^+^ tissue. **(A, B)** Signal-to-Noise Ratio (SNR) plots for AOIs where every point denotes a protein from the GeoMx Human Protein Assay for nCounter (Cell Death, Immune Activation Status, or Immune Cell Profiling panels) and the corresponding gene from the GeoMx Whole Transcriptome Atlas (WTA). Correlation between normalized expression intensities and normalized probe quantifications plotted for AOIs from CMV^+^ samples and CMV^-^ samples separately. **(A)** CD45^+^ AOIs with Spearman correlation and *p*-values; **(B)** PanCK^+^ AOIs with Spearman correlation and *p*-values. **(C, D)** Differential expression log2 fold change values for the spatial transcriptomics and spatial proteomics to identify agreement or changes in expression based on spatial modality—positive fold changes represent upregulation in CMV^+^ tissue. Green points represent *p*-value significant genes with agreement; red points represent *p*-value significant genes with disagreement between modalities.

## 4 Discussion

In this exploratory study, we used spatial transcriptomic and proteomic profiling to characterize CMV-associated changes in immune and trophoblast populations in the placenta. CMV^+^ placentae showed compartment specific alterations in immune activation and cell death pathways, suggesting that infection reshapes both maternal immune and trophoblast responses at the maternal fetal interface. Together, these findings identify candidate pathways through which CMV may promote persistence and vertical transmission.

In CD45+ immune cells, CMV infection was associated with altered apoptotic, proliferative, and immune signaling pathways. Suppression of death receptor signaling, including a decrease in CD95/Fas expression, is consistent with established CMV mechanisms that inhibit caspase-dependent apoptosis and promote survival of infected cells (21, 22). Other transcriptional changes, including altered NASP, FBLN1 and EBI3 expression, suggest further disruption of pathways regulating cell survival. Proliferation and antiviral immunity (23–29).

At the protein level, increased CD27 together with reduced CD45RO and several cell death associated proteins indicates a complex alteration of immune activation and differentiation rather than uniformed immune suppression (30, 31). These observations are consistent with the ability of CMV to modify T cell signaling and survival, although the functional consequences of these changes in placental cells require further study.

Trophoblast cells also demonstrated substantial alterations in in cell death and survival pathways. As appropriate trophoblast turnover is required for normal development and function of the placenta, disruption of these pathways could contribute to placental dysfunction associated with congenital CMV (32–37). Reduced expression of Granzyme A (GZMA) and several apoptosis associated proteins further suggest that CMV exposure may modify mechanisms involved in removal of infected cells (38, 39). Taken together, immune cell and trophoblast findings support that CMV simultaneously alters antiviral immune responses and trophoblast survival, potentially creating a placental environment permissive to viral persistence.

Immunohistochemical analysis further supported dysregulation of apoptotic pathways. CMV+ tissues contained more CC3 positive cells and fewer BCL2 positive cells than controls, with CC3 staining occurring predominately within the villi, while the STB layer remained largely unstained. This is consistent with the finding that multinucleated STBs do not engage in caspase-mediated apoptosis like CTBs do (40). These spatial differences emphasize that CMV associated effects on cell death may vary across cell populations and compartments. Additional studies will be required to determine how these findings relate to previously described CMV mechanisms that regulate caspase activation and BCL2-family proteins (41–45).

Our study has several limitations. Mostly importantly, the small sample size limits statistical power and generalizability, and the cohort likely overrepresents clinically apparent congenital infection because routine CMV screening was not available. Long-term infant outcomes were also unavailable, preventing assessment of relationships between placental molecular changes and disease severity. Additionally, the GeoMx protein panel provided targeted rather than comprehensive proteomic coverage, and functional experiments were beyond the scope of this study. Accordingly, these results should be considered hypothesis generating. Larger, prospective cohorts incorporating function validation and spatial localization of CMV antigen and RNA will be important for confirming these findings.

In conclusion, CMV infection was associated with spatially distinct alterations in immune activation, cell death and survival pathways in immune and trophoblast populations in the placenta. These findings provide preliminary insight into how CMV may reshape the placental microenvironment and identify candidate pathways for further investigation into the mechanisms of viral persistence and vertical transmission.

## Data Availability

Spatial transcriptomics data presented in this manuscript can be accessed on the NCBI GEO platform under GSE282298.
