# Supplementary material for: Spatial analysis of placentae during congenital cytomegalovirus infection suggests distinct cellular profiles in immune and trophoblast cells: an exploratory study
Source: Front Immunol. 2026 Oct 12;17:1885923. doi: 10.3389/fimmu.2026.1885923 (PMC13696421; doi:10.3389/fimmu.2026.1885923)
Supplement: Supplementary file 1 [file Table1.docx]

**SUPPLEMENTARY TABLE AND FIGURES**

**Supplemental Table 1.** CMV DNA Detection and Viral Load. NRAS qPCR analysis completed for human genomic DNA, standard and positive CMV control quantified as expected for CMV (UL83).

| Sample Type | ID no. | Date Tested | Cp UL83 (avg.) | Copies/uL  UL83 | UL pos. reps. | CP NRAS  (avg.) | Pg/uL  NRAS  (avg.) | Copies/ug |
| --- | --- | --- | --- | --- | --- | --- | --- | --- |
| Test | Mayo 1 | 01.27.2023 | **No amp.** |  | 0 of 3 | 24.91 | 1.10×10^4^ | **Negative** |
| Test | Mayo 2 | 01.27.2023 | **No amp.** |  | 0 of 3 | 25.21 | 8.96×10^3^ | **Negative** |
| Test | Mayo 3 | 01.27.2023 | **No amp.** |  | 0 of 3 | 25.38 | 8.01×10^3^ | **Negative** |
| Test | Mayo 4 | 01.27.2023 | **No amp.** |  | 0 of 3 | 26.62 | 3.43×10^3^ | **Negative** |
| Test | Mayo 5 | 01.27.2023 | **No amp.** |  | 0 of 3 | 25.79 | 6.06×10^3^ | **Negative** |
| Test | Mayo 6 | 01.27.2023 | **No amp.** |  | 0 of 3 | 25.38 | 7.97×10^3^ | **Negative** |
| Test | Mayo 7 | 01.27.2023 | 40.00 | 1.45×10^-1^ | 3 of 3 | 25.90 | 5.62×10^3^ | 2.58×10^1^ |
| Test | Mayo 8 | 01.27.2023 | 40.00 | 1.45×10^-1^ | 3 of 3 | 26.14 | 4.76×10^3^ | 3.05×10^1^ |
| Test | Mayo 9 | 01.27.2023 | 40.00 | 1.45×10^-1^ | 3 of 3 | 25.71 | 6.37×10^3^ | 2.28×10^1^ |
| Test | Mayo 10 | 01.27.2023 | 40.00 | 1.45×10^-1^ | 3 of 3 | 26.93 | 2.79×10^3^ | 5.20×10^1^ |
| Test | Mayo 11 | 01.27.2023 | 40.00 | 1.45×10^-1^ | 3 of 3 | 25.13 | 9.46×10^3^ | 1.53×10^1^ |
| Test | Mayo 12 | 01.27.2023 | 32.29 | 4.28×10^1^ | 3 of 3 | 26.62 | 3.42×10^3^ | 1.25×10^1^ |
| Neg. | Mayo 69 | 01.27.2023 | **No amp.** |  | 0 of 3 | 24.29 | 1.68×10^4^ | Negative |
| Neg. | Mayo 84 | 01.27.2023 | **No amp.** |  | 0 of 3 | 23.80 | 2.34×10^4^ | Negative |
| + CMV (std.) | Pos. ctl. (std) 1EE5 | 01.27.2023 | 20.55 |  |  | **No amp.** |  |  |
| + CMV | Pos. ctl. 1EE3 | 01.27.2023 | 27.13 | 1.00 ×10^5^ |  | **No amp.** |  |  |
| hgDNA (std.) | hgDNA (std.) 200pg/uL | 01.27.2023 | **No amp.** | 1.36×10^3^ |  | 30.79 | 2.00x10^2^ |  |

**Supplemental Figure 1.** Representative image showing morphology of the CMV^+^ placenta compared to control. A) Hematoxylin and Eosin staining of CMV^-^ placenta (top row) versus CMV^+^ placenta (bottom row), villi (left), decidua (right). B) IHC staining for CMV protein expression in the villi.

**Supplemental Figure 2.** Representative graph of the normalized read depth of transcriptomic reads for each AOI-type (i.e., CD45 or pan-cytokeratin). The provided histogram depicts the number of samples per bin (bin width = 2) within either CD45+ AOIs or PanCK+ AOIs. There were 145 AOIs sampled; minimum mean read depth was 13.7; maximum mean read depth was 45.4; 95% of samples have <120 mean read depth.

 

**Supplemental Figure 3.** Principal component analysis of the CMV GeoMx transcriptomic data after normalization. Scatterplot depicts separation between CMV^+^ and CMV^-^ samples along PC2. Less separation based on AOI-type (i.e., CD45^+^ or PanCK^+^ segment). "%" Annotations on x and y-axes denote the percent of explained variance per principal component. There were 5430 targets which were investigated for the 145 AOIs.

**Supplemental Figure 4.** Principal component analysis of the CMV GeoMx proteomic data after normalization. Scatterplot depicts separation between CMV^+^ and CMV^-^ samples along PC2. Less separation based on AOI-type (i.e., CD45^+^ or PanCK^+^ segment). "%" Annotations on x and y-axes denote the percent of explained variance per principal component. There were 49 probes which were investigated for 143 AOIs.

**Supplemental Figure 5.** Log2 signal-to-noise ratio for normalized intensities per marker. Negative control probes (i.e., MsIgG2a, MsIgG1, RbIgG) plotted at left. Boxplots of distributions provided as well as every AOI as points per marker. Values with mean at 0 represent proteins which have signal equivalent to mean background. Dotted line provided for 2-fold increase in expression over the mean background.
